# M2 Macrophages Mediate the Resistance of Gastric Adenocarcinoma Cells to 5-Fluorouracil through the Expression of Integrin *β*3, Focal Adhesion Kinase, and Cofilin

**DOI:** 10.1155/2020/1731457

**Published:** 2020-11-25

**Authors:** Daniel Ngabire, Irvine Niyonizigiye, Maheshkumar Prakash Patil, Yeong-Ae Seong, Yong Bae Seo, Gun-Do Kim

**Affiliations:** ^1^Department of Microbiology, College of Natural Sciences, Pukyong National University, 45 Yongso-ro, Nam-gu, Busan 48513, Republic of Korea; ^2^Gene and Cell Therapy Research Center for Vessel-associated Diseases, School of Medicine, Pusan National University, Yangsan, Republic of Korea; ^3^Research Institute for Basic Sciences, Pukyong National University, 45 Yongso-ro, Nam-gu, Busan 48513, Republic of Korea; ^4^Institute for Marine Biotechnology, Pukyong National University, 45 Yongso-ro, Nam-gu, Busan 48513, Republic of Korea

## Abstract

Tumor microenvironment components dictate the growth and progression of various cancers. Tumor-associated macrophages are the most predominant cells in TME and play a major role in cancer invasiveness. Gastric cancer is one of the most common cancers in Asia, and recently, various cases of resistance to fluorouracil treatment have been reported. In this study, we investigated the role of alternatively activated macrophages in the resistance of AGS gastric cancer cells to fluorouracil. THP-1 cells were polarized using recombinant human IL-4, then were cocultured with AGS cells treated with fluorouracil. Cell viability, Western blot, immunofluorescence, and cell invasion were performed for this investigation. Our results demonstrated that polarized macrophages initiated the survival of treated AGS cells and induced the resistance in AGS by upregulating the expression of integrin *β*3, focal adhesion protein (FAK), and cofilin proteins. These results reveal that integrin *β*3, focal adhesion protein (FAK), and cofilin proteins are potential targets for the improvement of fluorouracil efficacy in gastric cancer treatment.

## 1. Introduction

The tumor microenvironment (TME) is composed of cancer cells and a high number of infiltrating stromal cells such as fibroblasts, endothelial cells, and immune cells. Tumor-associated macrophages (TAMs) are the most prominent immune cells in TME [1]. As a major component of tumor immunity, macrophages can inhibit or promote the proliferation of cancer cells and tissue repair [2]. Two phenotypes of macrophages are now known in the tumor microenvironment: Classically activated macrophages (or M1-type macrophages) produce molecules that prevent the proliferation of surrounding cells and alternatively activated macrophages (or M2-type macrophages) release cytokines that favor the proliferation of tumor cells [3, 4].

Polarized macrophages produce different types of cytokines. M2 macrophages promote the expansion of tumors by enhancing proliferation [5], metastasis [6], angiogenesis [7], lymphangiogenesis [8], and immunosuppression [9]. These factors allow us to conclude that tumor-associated macrophages (TAMs) represent an efficient target for the treatment of different types of tumors [10] as all the mechanisms involved in the role of TAMs during chemotherapy treatment of gastric adenocarcinoma are still not fully understood.

Fluorouracil (5FU) is an anticancer agent used in the treatment of various cancers including gastric cancer. The cytotoxicity of 5FU is related to its ability to inhibit the synthesis of nucleotides. It is used in the treatment of various cancers alone or in combination with other chemotherapeutic agents [11, 12].

In recent years, various studies have reported the increase of resistance of gastric cancer tumors against 5FU. Different cell processes can mediate the chemoresistance such as the inhibition of apoptosis or migratory and invasiveness proprieties. Epithelial-mesenchymal transition (EMT) induces cancer cell migration and invasion of cancer cells which leads to metastasis [13–15]. During the EMT, E-cadherin gradually decreases while vimentin increases [16, 17]. E-cadherin is a cell adhesion protein present in epithelial cells and is involved in the inhibition of cancer cells migration. Vimentin is an intermediate filament highly expressed in mesenchymal cells and is used as a marker for metastatic cancer cells. Research publications have also demonstrated that cellular levels of some integrins, such as *α*v and *β*3, can assist in the initiation of metastasis in cancers, including gastric cancer [18–20]. Focal adhesion kinase (FAK) participates in the focal adhesion dynamic. FAK facilitates the regulation of cell adhesion and modulates migration signals in cancer cells. FAK takes part in the involvement of integrins and mounting of focal adhesions (FA) through catalyzing several intracellular pathways and mediates cell behavior. In addition, cofilin, a protein of cell cytoskeleton, contributes significantly to the migration and invasion of cancer cells during metastasis. By severing actin filaments, cofilin creates ends required for lamellipodium extension during cell migration [21].

In this study, we investigated the role of M2 macrophages in the resistance of AGS cells to treatment with 5FU.

## 2. Materials and Methods

### 2.1. Cell Viability Assay

AGS cells (ATCC, Rockville, USA) and THP-1 cells (ATCC, Rockville, USA) were plated in 96-well plates at a final concentration of 1 × 10^4^ cells/well for 24 h before the experiments. Cells were then treated with various concentrations of 5FU for 24 h with (or without) conditioned media (CM) from M1 and M2 macrophages for 24 h. After treatment, the media in the wells were removed and replaced by fresh prewarmed media. WST-1 was added to each well and the plates were incubated at 37°C for an additional period of 4 h. The absorbance was measured using a Multiplate reader at a wavelength of 490 nm.

### 2.2. Macrophage Polarization

For M1-like and M2-like phenotypes, THP-1 cells were plated into 6-well plates or 6-well plate inserts and incubated for 24 h at 37°C. Initially, phorbol-12-myristate-13-acetate (PMA; 320 nM) was added to THP1 cells for differentiation. Following 24 h, THP1 cells were induced into the M0 phenotype. To generate M1-like macrophages, THP-1 macrophages were activated with LPS (1 *μ*g/ml) for 24 h. As for M2-like macrophages, THP-1 cells activated IL-4 (20 ng/ml), conditioned medium (CM) from AGS cells, or the inserts containing previously plated THP-1 cells were placed in wells containing AGS cells (coculture). Conditioned media from THP-1 cells or AGS cells were collected as follows: for AGS cells, AGS cells were cultured in T75 flasks till they reach 70% of confluency, while for THP-1 cells, cells were plated in petri dishes then polarized for 24 h. The supernatant media were centrifuged at 1000 rpm for 5 min and filtrated through a 0.2 *μ*M pore size filter. The obtained conditioned media were stored at -20°C.

### 2.3. Coculture Experiments

THP-1 cells were plated on the upper compartment of 0.4 *μ*m pore size Transwell of 6-well plates in 1 ml of RPMI complete medium, while AGS cells (3 × 10^6^ cells) were plated in the lower chamber. Initially, AGS cells and THP-1 cells were cultured in separate plates. THP-1 cells were polarized in M2 macrophages using IL-4 and CM from AGS cells for 24 h like described above and after polarization, the inserts were transferred on the plate containing AGS cells. Before the transfer of inserts in the wells containing AGS cells, AGS cells were treated with 10 *μ*g/ml of 5FU. The plates were then incubated for 18 h and after treatment further analyses were conducted.

### 2.4. NO Assay

THP-1 cells were plated in 24-well plates and incubated at 37°C for 24 h. After 24 h of incubation, THP-1 cells were activated with LPS (1 *μ*g/ml), IL-4 (20 ng/ml), conditioned media (40% of CM and 60% of fresh media), and coculture (0.4 *μ*m pore size inserts of 24 well plate) for an additional 24 h. The levels of NO were calculated using the Griess reagent assay. Briefly, 24 h after treatment with LPS, IL-4, CM, and coculture, 100 *μ*l of culture media supernatant from each sample was mixed with an equal volume of the Griess reagent. After 15 min of incubation, the absorbance values were obtained by using an ELISA microplate reader at 540 nm. The levels of nitrite contained in the samples were determined by comparing with the standard concentrations of sodium nitrite diluted in RPMI.

### 2.5. Western Blot

For the protein expression, THP-1 cells and AGS cells were cultured as described above. After activation or treatment, the cells were collected by trypsinization and proteins were extracted using a cell lysis buffer. Samples were loaded into 12% of acrylamide gel. After electrophoresis, the proteins in the gels were transferred onto a PVDF membrane (Bio-Rad, Hercules, CA, USA). Once the transfer was completed, the membranes were blocked for 1 h with 5% BSA in TBS 0.1% Tween-20 (TBS-T). The membranes were then incubated with IL-1*β*, IL-6, iNOS, IL-10, Arg-1, MARCO, pPI3K, pAkt, p-p65 NF-*κ*B, MMP-2, MMP-9, integrin *β*3, ZEB-1, Snail, pFAK, vimentin, E-cadherin, cofilin, and GAPDH first antibody diluted 1 : 1000 in PBST at 4°C for overnight. The next day, the membranes were washed with PBST and secondary antibody (goat anti-rabbit IgG-HRP, Sc-2004; Santa Cruz Biotechnology, Inc., Dallas, TX, USA) diluted 1 : 1,000 in PBST was added to the membranes for 1 h at room temperature.

### 2.6. Cell Invasion Assay

For cell invasion, Transwell (Costar, Lowel, MA) with 8 *μ*m polycarbonate filter-membranes in 24-well plates was used following the manufacturer's instructions. The experiments were conducted as previously described [22] with minor modifications. Briefly, the lower side of the filter-membrane was coated with 100 *μ*l of Matrigel (Corning, Bedford, MA). AGS cells were seeded in the inserts coated with Matrigel and incubated for 24 h at 37°C. After 24 h incubation, AGS cells were treated (or not) with 10 *μ*g/ml of 5FU diluted in media without FBS, and then the inserts were placed into wells containing (or not) polarized M2 macrophages. The cells were incubated for further 24 h at 37°C and after incubation; cells that had not crossed the membrane on the upper side were scrubbed off with a cotton stick. Migrated cells on the other were fixed with ice-cold methanol then stained with 0.5% crystal violet (Sigma Chemicals, St. Louis, MO) for 20 min. After washing with PBS, the cells were observed using an inverse microscope (magnification, ×100).

### 2.7. Immunofluorescence

Immunohistochemistry analysis was performed as described previously [22] with minor modifications. Briefly, THP-1 cells and AGS cells were plated on cover-glass bottom dishes, incubated at 37°C. THP-1 cells, after polarization with LPS and IL-4 for 24 h, were first stained at room temperature with 1 *μ*g/ml of diamidino-2-phenylindole dye (DAPI) for 20 min. After DAPI, cells were fixed in 4% formaldehyde at room temperature for 15 min and then stained with F-actin for 20 min at room temperature. For AGS cells, cells were stained with DAPI then fixed in 4% formaldehyde at room temperature for 15 min and blocked for 1 h in a blocking solution, including 5% rabbit and mouse normal serums with 0.3% Triton X-100. After fixation and blocking, primary antibodies (cofilin, E-cadherin, *β*-actin) were added to the cover-glass dishes for 3 h. Once washing with PBS buffer was completed, the cells were incubated with anti-mouse IgG (H+L), F(ab')2 fragment (Alexa Fluor® 555 Conjugate), or anti-rabbit IgG (H+L), F(ab')2 fragment (Alexa Fluor® 488 Conjugate) for 1 h. Cells were mounted on the slide with Prolong Gold Antifade Reagent, and images were obtained using a ZEISS LSM 710 confocal laser scanning microscope (Carl Zeiss, Jena, Germany).

### 2.8. Statistical Analysis

Our data are presented as mean ± standard deviation (SD) of 3 separate sets of data for each experiment. The mean values of the control were compared with the mean values of each treatment group by two-way ANOVA using the statistical software GraphPad Prism 7 (GraphPad Software Inc., La Jolla, CA, USA), and a statistically significant difference was set at ∗∗∗*P* < 0.001 and ∗∗∗∗*P* < 0.0001.

## 3. Results

### 3.1. LPS and IL-4 Successfully Polarized THP-1 Cells into M1 and M2 Macrophages, Respectively

**Figure 1:**
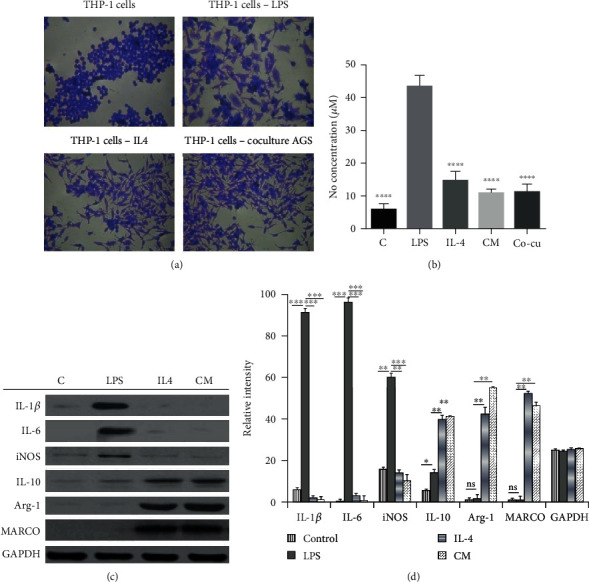
Polarization of THP-1 cells into M1 and M2 macrophages with LPS, IL-4, conditioned media, and coculture. (a) Crystal violet staining of polarized THP-1 cells. (b) DAPI and F-actin staining of polarized THP-1 cells. (b) NO assay of polarized THP-1 cells. (c) Western blot analysis of polarized THP-1 cells. In these experiments, THP-1 cells were plated in 24-well plates and 24-well plate inserts of 0.4 *μ*m pore size. The plates were incubated for 24 h at 37°C. After 24 h of incubation, THP-1 cells were stimulated with LPS (1 *μ*g/ml), IL-4 (40 ng/ml), and conditioned medium (CM) from AGS cells, and finally, inserts containing THP-1 cells were added to well containing AGS cells, and the plates were incubated for further 24 h and further analyses were conducted. (d) Quantification of western blot bands. Statistically significant difference was set at ∗*P* < 0.05, ∗∗*P* < 0.01, ∗∗∗*P* < 0.001, and ∗∗∗∗*P* < 0.0001.

We investigated the in vitro polarization of THP-1 cells. Macrophages can be classically (M1) or alternatively (M2) activated. M1 macrophages are characterized by the production of inflammatory cytokines (IL-1*β*, IL-6, iNOS) while M2 macrophages produce commonly IL-10, Arginase-1, and MARCO. To obtain M1-like and M2-like macrophages, THP-1 cells were stimulated separately with LPS and IL-4, respectively, for 18-24 h. As shown in Figures 1(a) and 1(b), we observed a significant difference in shape between M1-like and M2-like macrophages. M1-like macrophages presented an irregular shape with multiple pseudopods while M2-like macrophages had an elongated shape without pseudopods. The NO assay (Figure 1(c)) revealed that M1-like macrophages secreted high amounts of nitric oxide (NO) while M2-like macrophages had low levels of NO. Additionally, Western blot results (Figure 1(d)) revealed that M1-like macrophages produced higher levels of IL-1*β*, IL-6, and iNOS while M2-like macrophages produced higher levels of Arg-1 and expressed the MARCO receptor. These results confirmed that we were able to polarize THP-1 cells into classically activated macrophages (M1) and alternatively activated macrophages (M2 macrophages). In addition to LPS and IL-4 activation, THP-1 activated with conditioned medium (CM) from gastric adenocarcinoma (AGS) cells were morphologically similar to M2 macrophages. The same results were observed in a coculture of AGS cells and THP-1 cells.

### 3.2. M2 Macrophages Induced Cell Survival in AGS Cells through the PI3K/Akt/NF-*κ*B Pathway

**Figure 2:**
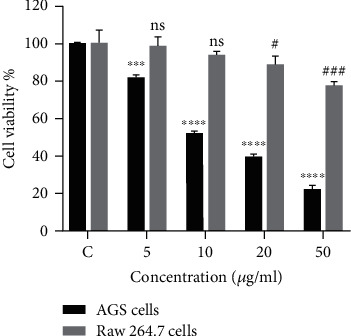
Cell viability of AGS cells and THP-1 cells treated with 5FU. AGS cells and THP-1 cells were cultured in RPMI media and incubated for 24 h at 37°C. After 24 h of incubation, cells were treated with various concentrations of 5FU and incubated for further 24 h. After treatment, the media in the plate were removed and replaced with fresh media, and then 10 *μ*l of WST-1 solution was added in each well, and the plate was incubated for further 4 h at 37°C. Absorbance was obtained using the ELISA microplate reader at 490 nm wavelength. Statistically significant difference was set at ^#^*P* < 0.05, ^###^*P* < 0.001, ∗∗∗*P* < 0.001, and ∗∗∗∗*P* < 0.0001.

**Figure 3:**
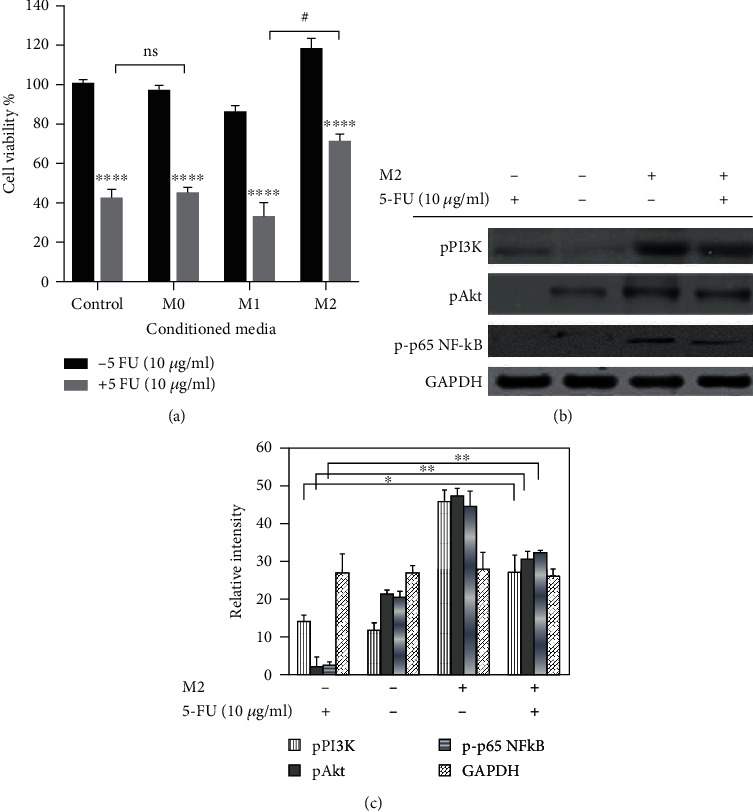
(a) Cell viability of AGS cells treated with 5FU with conditioned media from polarized macrophages. AGS cells were plated in 96-well plates and incubated for 24 h at 37°C. After 24 h of incubation, the media were replaced with a mixture of fresh media and conditioned media (CM) from macrophages to a final ratio of 60 : 40, respectively (control = no CM, M0 = CM from PMA-differentiated THP-1 cells, M1 = CM from THP-1 cells activated with LPS, and M2 = CM of THP-1 cells activated with IL-4). 5FU was simultaneously added to the cell to a final concentration of 10 *μ*g/ml and the plates were incubated for 24 h. After treatment, the media in the plate was removed and replaced with fresh media, and then 10 *μ*l of WST-1 solution was added in each well, and the plate was incubated for further 4 h at 37°C. Absorbance was obtained using the ELISA microplate reader at 490 nm wavelength. (b) Proliferation of AGS cells cocultured with M2 macrophages. AGS cells were plated in 6-well plates and incubated for 24 h at 37°C. After 24 h of incubation, AGS cells were treated with 10 *μ*g/ml of 5FU, and then polarized THP-1-M2 macrophages cultured in 6-well plate inserts of 0.4 *μ*m pore size were added to the AGS wells and incubated for additional 24 h. After treatment, proteins were extracted from AGS cells and separated by Western blot. (c) Quantification of western blot bands. Statistically significant difference was set at ^#^*P* < 0.05, ∗*P* < 0.05, ∗∗*P* < 0.01.

M2 macrophages reportedly induce proliferation of cancer cells in the tumor microenvironment. We investigated if M2 macrophages played a role in the resistance of AGS cells to 5FU treatment. AGS cells and THP-1 cells were treated with increasing concentrations of 5FU and after the analysis of obtained results; we decided to use 10 *μ*g/ml concentration for further experiments (Figure 2). To investigate if activated macrophages have a contribution in the resistance of AGS cells to 5FU, AGS cells were plated in 96 wells for 24 h; then after 24 h, the medium was replaced with conditioned media (CM) from MO (nonactivated macrophages), from M1 macrophages (LPS-activated macrophages) and M2 macrophages (IL-4 activated macrophages) mixed (or not) with 5FU. The results presented in Figure 3(a)) show that CM from M2 macrophages increased the proliferation of AGS cells in both nontreated and treated scenario with 5FU while CM from M1 macrophages combined 5FU provided the highest anticancer effect. We further investigated if the PI3K/Akt/NF-*κ*B pathway played a role in the survival of AGS cells and we uncovered that 5FU-treated AGS cells cocultured with M2 macrophages expressed higher levels of pPI3K, pAkt, and p-p65 NF-*κ*B proteins (Figure 3(b)). These results allowed us to conclude that M2 macrophages probably mediated the survival of 5FU-treated AGS cells through the activation of the PI3K/Akt/NF-*κ*B pathway.

### 3.3. M2 Macrophages Induced Cell Invasion in AGS Cells

**Figure 4:**
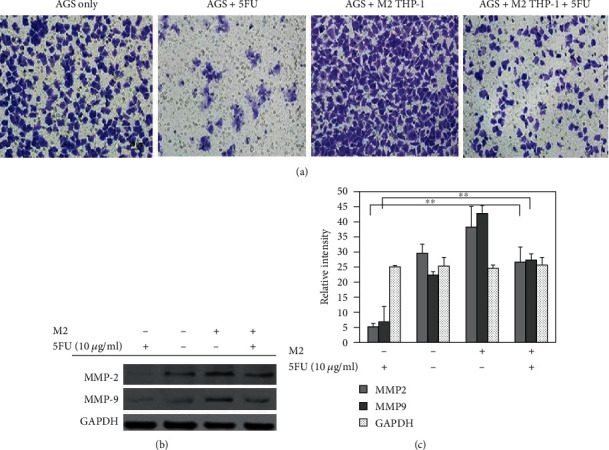
Cell migration and invasion of AGS cells. (a) AGS cells were plated in 0.8 *μ*m pore size Transwell and incubated for 24 h. After 24 h incubation, AGS cells were treated with 10 *μ*g/ml of 5FU in FBS free media, and the inserts were added in wells containing polarized THP-1-M2 macrophages or fresh media and incubated for further 24 h. After 24 h, nonmigrating cells on the upper side of the membrane were removed and migrated cells on the lower side of the membrane were fixed with ice-cold methanol. Fixed membranes were stained with 0.5% crystal violet. (b) Western blot analysis of MMP-2 and MMP-9 proteins. (c) Quantification of western blot bands. Statistically significant difference was set at ∗∗*P* < 0.01.

**Figure 5:**
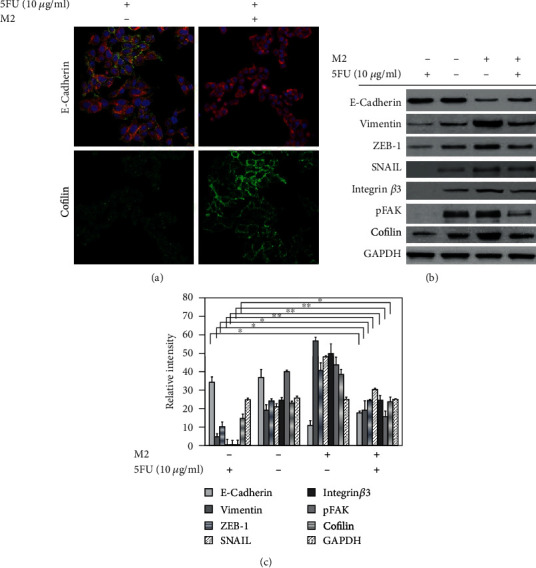
(a) E-cadherin and cofilin expression in AGS. AGS cells were plated in 6-well plates containing cover glass for 24 h. After 24 h of incubation, AGS cells were treated with 10 *μ*g/ml of 5FU and the inserts containing polarized THP-1-M2 macrophages were added to the AGS wells. AGS cells were then stained with DAPI and with E-cadherin, *β*-actin, and cofilin antibodies. The stained cells were observed under a confocal microscope. (b) Western blot analysis of EMT factors. Western blot analysis of E-cadherin, vimentin, ZEB-1, Snail, integrin *β*3, pFAK, and cofilin expression. (c) Quantification of western blot bands. Statistically significant difference was set at ∗*P* < 0.05, ∗∗*P* < 0.01.

EMT is a transition of cells from epithelial phenotype to mesenchymal is one of the initial steps after the ECM degradation that allows cancer cells to reach blood circulation. The matrix metalloproteinases (MMPs) play a crucial function in tissue remodeling by degrading the ECM. MMP-2 and MMP-9 break down the ECM, and the resulted components interact with integrins to activate further cell processes. As shown in Figure 4(a), AGS cells cocultured with M2 macrophages were able to migrate despite the treatment with 5FU. Western blot results (Figure 4(b)) revealed a higher expression of MMP-2 and MMP-9 in treated AGS cells cocultured with M2 macrophages which suggest that they play a crucial role in the invasion of treated AGS cells. EMT is characterized by low levels of E-cadherin and high levels of vimentin. ZEB-1 and Snail transcription factors repress E-cadherin and induce EMT. Additionally, cofilin and FAK protein play a major role in cancer cell invasiveness. We investigated the expression of EMT proteins and transcription factors in 5FU-treated AGS cells cocultured with M2 macrophages. Our results demonstrated the loss of E-cadherin expression and upregulation of vimentin, ZEB-1, Snail, integrin *β*3, pFAK, and cofilin. (Figures 5(a) and 5(b)).

## 4. Discussion

The progression of tumors is controlled by the interaction between tumor cells and other cell types within the TME. Macrophages are known to be polarized into M1 and M2 phenotypes in the TME. We were successfully able to polarize THP-1 cells into M1 and M2 macrophages using LPS and IL-4, respectively, as stimulants or activators. Significant morphological differences were observed between obtained M1 and M2 macrophages. Like already reported by previous studies, M1 macrophages produced high levels of nitric oxide compared to M2 macrophages and Western blot results confirmed that M1 macrophages produced high levels of inflammatory cytokines (IL-1*β*, IL-6, and iNOS) while M2 macrophages produced high levels of Arg-1 and expressed the mannose receptor MARCO (Figure 1(d)). THP-1-M2 macrophages increased the proliferation of AGS cells in both treated and not treated cases with 5FU. The results of cell viability showed that the percentage of AGS cells cultured with conditioned media from M2 macrophages was higher compared to control AGS cells cultured just with fresh media or media from M1 macrophages. The treatment of AGS cells with 5FU and the addition of M2 media also increased AGS cell proliferation compared to 5FU-treated AGS cells without conditioned media (Figure 3(a)). From these results, we were able to establish that M2 macrophages induced the resistance of AGS against 5FU and promoted cell survival. The PI3K/Akt/NF-*κ*B pathway has been reported in various studies to play a crucial role in cell proliferation and cell survival. Western blot results of phosphorylated forms of PI3K and Akt showed a significant increase in the expression of PI3K, Akt, and NF-*κ*B proteins (Figure 3(b)); therefore, we were able to determine that the survival of 5FU-treated AGS cultured with conditioned media from THP-1-M2 macrophages was at least mediated partially by the PI3K/Akt pathway.

Metastasis and malignancy are the first and second main causes of cancer-related deaths, respectively [23]. In recent decades, various studies have highlighted how macrophages play a major role in tumor growth, progression, and metastasis [5, 24]. In gastric adenocarcinoma, the role of TAMs as tumor-promoters is further validated by clinical studies revealing a connection between the presence of a high number of macrophages in tumor tissue and poor patient prognosis; however, the roles of TAMs in chemotherapy and chemoresistance are not well-understood to date [25].

The MMP enzymes are a family of proteases that can disrupt the extracellular matrix (ECM) proteins, such as collagen, laminin, and proteoglycan [26–28]. We conducted a cell invasion assay in AGS cells treated or not with 5FU in a coculture setup using M2 macrophages as a chemoattractant. The results showed that 5FU-treated AGS cells cocultured with M2 macrophages migrated more through the membrane coated with the Matrigel compared to 5FU-treated AGS cells only (Figure 4(a)). To further understand, we analyzed the expression of metalloprotease enzyme and western blot results revealed that MMP-2 and MMP-9 proteases were upregulated in 5FU-treated AGS cells cocultured with M2 macrophages; therefore, we can conclude that M2 macrophages can increase the invasion of AGS cells despite the presence of 5FU (Figure 4(b)). Cofilin is a protein that binds to actin molecules and that has an important role in the regulation of actin dynamics [29, 30]. The cofilin-mediated severing of actin filaments at the leading edge of motile cells controls the formation of lamellipodia, which is essential cancer cell metastasis [31, 32]. We analyzed the expression of cofilin in 5FU-treated AGS cells and nontreated AGS cells both cocultured with M2 macrophages and revealed that cofilin was highly expressed in 5FU-treated AGS cells cocultured with M2 macrophages (Figure 5(a)). These results were confirmed by Western blot analysis (Figure 5(b)); therefore, we can establish that the activation of cofilin is part of the mechanisms involved in cell migration of 5FU-treated AGS induced by coculture with M2 macrophages.

EMT is one of the multiple factors that play a crucial role in metastasis and had attracted considerable attention in cancer research [15, 33–35]. Cancer progression and aggressive behaviors are facilitated by the evolution of EMT in the tumor microenvironment through the increase of cell migration and invasion [35–37]. Our immunofluorescence results showed that E-cadherin expression was lost in 5FU-treated AGS cells cultured with M2 macrophages (Figure 5(a)), and these results were confirmed by Western blot analysis of E-cadherin protein expression (Figure 5(b)).

Integrins are a family of cell receptors that bind to ECM proteins, and the downstream pathways include the phosphorylation of FAK [38, 39]. FAK protein drives cell survival and differentiation including the growth of tumors, migration, and tissue remodeling before metastasis. The activation of FAK triggers a series of biological processes that include cell attachment, migration, invasion, proliferation, and survival [40]. FAK has been demonstrated to be an efficient target for cancer treatment, and currently, there are ongoing clinical trials. In the present study, we investigated the hypothesis of a potential role of integrin *β*3 and its associated complex in the resistance of AGS cells to 5FU when cocultured with M2 macrophages. Our results determined that 5FU-treated AGS cocultured with THP-1-M2 macrophages expressed increases levels of integrin *β*3 and pFAK. In addition to these results, our study supported previously published data that demonstrated the beneficial role of M1 macrophages in cancer treatment. There are many agents currently being evaluated for their potential to switch macrophages phenotype from M2 to M1. Our findings still need to be confirmed in other gastric cell lines and in in vivo models, but we strongly believe that the combination of 5FU with drugs that can suppress M2 macrophages or induce a conversion from M2 to M1 phenotype might significantly improve the treatment success rate in gastric cancer patients. FAK, cofilin, and integrin beta-3 should be more deeply investigated to fully uncover their role in the resistance of gastric cancer to 5FU treatment.

## Data Availability

Available on reasonable request.
